# Non-Random Pattern of Integration for Epstein-Barr Virus with Preference for Gene-Poor Genomic Chromosomal Regions into the Genome of Burkitt Lymphoma Cell Lines

**DOI:** 10.3390/v14010086

**Published:** 2022-01-04

**Authors:** Snjezana Janjetovic, Juliane Hinke, Saranya Balachandran, Nuray Akyüz, Petra Behrmann, Carsten Bokemeyer, Judith Dierlamm, Eva Maria Murga Penas

**Affiliations:** 1 Department of Oncology, Hematology and Bone Marrow Transplantation with Section Pneumology, University Clinic Hamburg-Eppendorf, 20251 Hamburg, Germany; s.janjetovic@uke.de (S.J.); j.hinke@gmx.de (J.H.); n.akyuez@uke.de (N.A.); pbehrmann@uke.de (P.B.); cbokemeyer@uke.de (C.B.); 2 Clinic of Hematology and Stem Cell Transplantation, HELIOS Clinic Berlin-Buch, 13125 Berlin, Germany; 3 Department for Psychiatry, Albertinen Hospital, 22459 Hamburg, Germany; 4 Institute of Human Genetics, Christian-Albrechts-University of Kiel and University Hospital Schleswig-Holstein, Campus Kiel, 24118 Kiel, Germany; sbalachandran@medgen.uni-kiel.de

**Keywords:** Epstein-Barr virus, integration sites, Burkitt lymphoma cell lines

## Abstract

Background: Epstein-Barr virus (EBV) is an oncogenic virus found in about 95% of endemic Burkitt lymphoma (BL) cases. In latently infected cells, EBV DNA is mostly maintained in episomal form, but it can also be integrated into the host genome, or both forms can coexist in the infected cells. Methods: In this study, we mapped the chromosomal integration sites of EBV (EBV-IS) into the genome of 21 EBV+ BL cell lines (BL-CL) using metaphase fluorescence in situ hybridization (FISH). The data were used to investigate the EBV-IS distribution pattern in BL-CL, its relation to the genome instability, and to assess its association to common fragile sites and episomes. Results: We detected a total of 459 EBV-IS integrated into multiple genome localizations with a preference for gene-poor chromosomes. We did not observe any preferential affinity of EBV to integrate into common and rare fragile sites or enrichment of EBV-IS at the chromosomal breakpoints of the BL-CL analyzed here, as other DNA viruses do. Conclusions: We identified a non-random integration pattern into 13 cytobands, of which eight overlap with the EBV-IS in EBV-transformed lymphoblastoid cell lines and with a preference for gene- and CpGs-poor G-positive cytobands. Moreover, it has been demonstrated that the episomal form of EBV interacts in a non-random manner with gene-poor and AT-rich regions in EBV+ cell lines, which may explain the observed affinity for G-positive cytobands in the EBV integration process. Our results provide new insights into the patterns of EBV integration in BL-CL at the chromosomal level, revealing an unexpected connection between the episomal and integrated forms of EBV.

## 1. Introduction

Epstein-Barr virus (EBV), a human gamma-1 herpesvirus, is widespread worldwide and is carried as a latent asymptomatic infection in the vast majority of individuals. Nevertheless, EBV has a powerful growth-transforming ability in cells of epithelial and lymphoid origin and is etiologically linked to a range of lymphoproliferative lesions, malignant lymphomas, and carcinomas [1]. Among the hematological malignancies, several entities of B- and T-cell neoplasias are linked to an EBV infection, i.e., Burkitt lymphoma (BL), natural killer T-cell lymphoma (NKTCL), systemic EBV+ T-cell lymphoma of childhood, Hodgkin lymphoma, and diffuse large B-cell lymphoma [2,3]. In addition, EBV infection is etiologically associated with southern China endemic nasopharyngeal carcinoma (NPC) [4].

BL is a highly aggressive mature B-cell lymphoid neoplasia [2]. The hallmark of this disease is the overexpression of *MYC*, commonly resulting from the translocation t (8;14) (q24; q32)/*MYC-IGH* or less commonly its variant translocations, t(2;8)(p12;q24)/*MYC*-*IGK* and t(8;22)(q24;q11)/*MYC-IGL* [5,6]. According to their clinical characteristics, the WHO classification of “Tumours of Haematopoietic and Lymphoid Tissue” distinguishes three different forms of BL: endemic BL (eBL), sporadic BL (sBL), and human immunodeficiency virus (HIV) associated BL [2]. The eBL mostly affects patients in the African malaria belt, where approximately 95% of BL cases carry the virus [6]. In HIV-associated BL, EBV infection is found in approximately 30–40% of patients [7]. In contrast, sBL is rarely associated with EBV (5–10%), although in some areas, such as Brazil, EBV-positive (EBV+) sBL cases can exceed 80% [7]. Hence, EBV is not crucial for the development of BL. Although, in EBV+ cases, the etiological role of EBV infection in the development of BL is supported by the finding that every tumor cell harbors a monoclonal EBV genome [7]. However, whether EBV infection precedes or follows the *MYC* translocation and which viral genes are involved at different stages of the BL pathogenesis is still unclear [3,8].

Typically, the EBV genome exists as extrachromosomal episomes in infected cells [9,10]. In addition to its episomal form, the EBV genome also integrates into the human genome, preferentially in gene-poor G-band-positive material [11,12,13]. However, whether the EBV integration follows a random or non-random distribution is still debatable. Up to now, EBV integration sites (EBV-IS) reported in 8 BL cell lines (BL-CLs), 24 EBV-transformed lymphoblastoid cell lines (LCL), and 1 Hodgkin lymphoma cell line have shown viral integration at different chromosomal loci (Table 1) [12,13,14,15,16,17,18,19,20,21,22,23,24,25,26,27].

In some BL-CL, the precise sites of integration could be characterized (Table 1). More recently, whole genome sequencing (WGS) has identified novel EBV-IS in the *NHEJ1* gene in NKTCL [28] integrating into the intronic regions of inflammatory-related genes and downstream of tumor-suppressor genes in NPC and gastric carcinoma [29]. These studies have also reported lower rates of EBV integration into the human genome than in other DNA viruses such as hepatitis B virus (HBV) and human papillomavirus (HPV) [28,29]. Despite the cytogenetic data that point to a non-random pattern of integration for EBV into the human genome [14], WGS studies have not found an occurrence of recurrent sites for EBV as it has been identified for other viruses such as HBV, so far [30,31]. To better understand the biological significance of EBV integration, we explore further cytogenetic sites of EBV-integration in 20 EBV+ BL-CL that were previously characterized karyotypically [32].

## 2. Materials and Methods

### 2.1. Cell Lines

EBV-integration sites (EBV-IS) in 20 EBV+ BL-CL were investigated in this study. Data of karyotypic characterization and authentication of the BL-CL have been previously analyzed in an exhaustive molecular cytogenetic study [32] and are summarized in Table S1 of Supplementary Materials. Culturing, harvesting, and fluorescence in situ hybridization (FISH) on the metaphase spreads of the BL-CLs were carried out according to standard methods as previously described [32].

### 2.2. Amplification and Detection of EBV DNA by PCR

The presence of the EBV genome in BL-CL was verified by PCR. Briefly, genomic DNA from BL-CLs was isolated using a QIAamp DNA Mini Kit (Qiagen, Hilden, Germany). Viral DNA was amplified by PCR using either Probe-1 or Probe-2 primers’ pairs (sequences previously published) [23] and DNA Polymerase AccuPrime Pfx (Invitrogen, Darmstadt, Germany) according to the supplier’s recommendations.

### 2.3. Assessment of the EBV-IS by FISH and Data Analysis

FISH for the detection of EBV-IS was performed using, as the EBV-DNA probe, a biotinylated 3000 bp fragment amplified by PCR with primers’ pair Probe-1 from 1.5 µg DNA of the EBV+ BL-CL Raji, as previously described [23]. Probe-1 encompasses the sequences of the largest repeat of EBV, IR1, also known as the BamHI W fragment (www.ncbi.nlm.nih.gov accessed on 3 January 2022; Acc. No. M15973.1), which is composed of about 6.6 copies of a 3 kb sequence [32] and therefore renders clearly visible hybridization signals.

**Figure 1:**
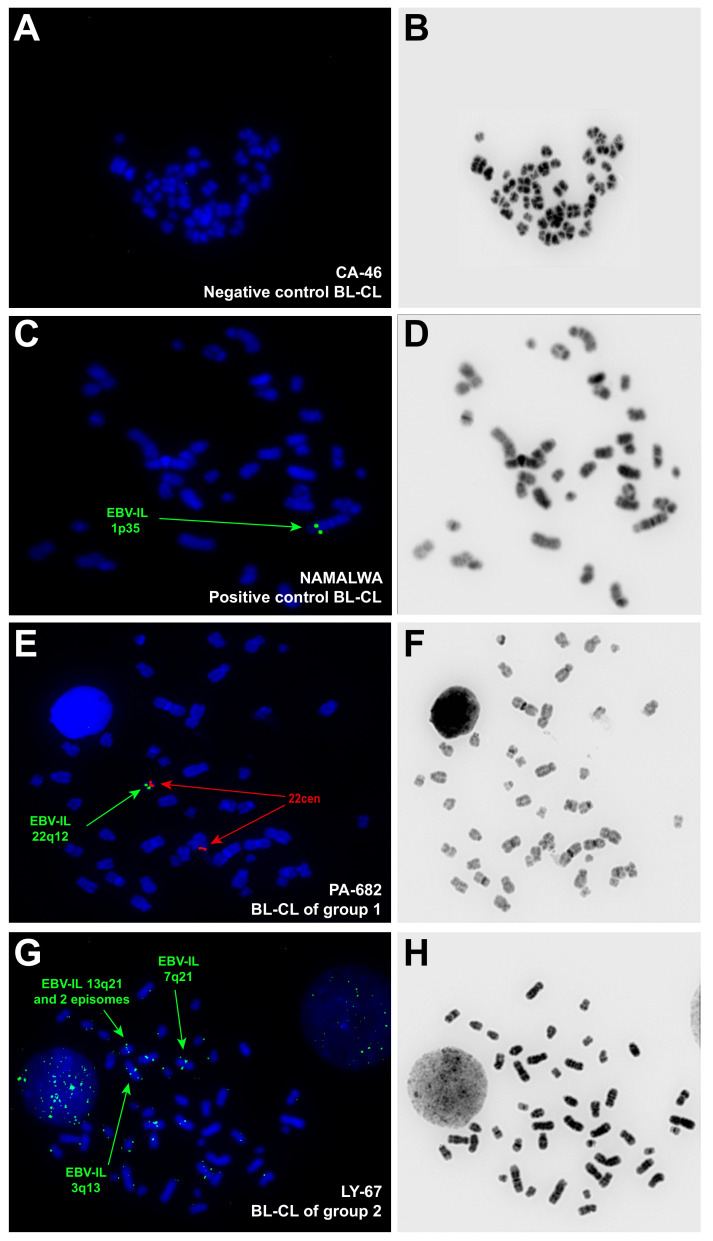
Representative images of EBV-IS using FISH on metaphases of BL-CL. The presence of symmetrical doublet hybridization signals at the same chromosomal loci of both sister chromatids represents the integration of EBV in the host genome, whereas single weak hybridization signals represent episomal viral locations. The inverted DAPI-counterstaining used for chromosomes assignment is also displayed (**B**,**D**,**F**,**H**). (**A**,**B**) EBV-negative BL-CL CA-46 was used as a negative control and showed, as expected, no hybridization signals of the EBV probe. (**C**,**D**) One EBV-IS located on 1p35, green hybridization signals highlighted with a green arrow, was visualized in the BL-CL Namalwa (group 1). (**E**,**F**) A unique EBV-IS on cytoband 22q12 was identified in PA-682 (group 1), green hybridization signals highlighted with a green arrow. A centromere probe for chromosome 22, red signals highlighted with a red arrow, was co-hybridized to confirm the localization of the integration of EBV on chromosome 22. (**G**,**H**) In BL-CL LY-67 (group 2) highly recurrent EBV-IL on 7q21 and 13q21, and non-recurrent IL on 3q13 is highlighted with green arrows.

Direct Sanger-sequencing for confirmation of the specificity of the amplified products (data not shown) and labeling with biotin-dUTP by nick-translation were performed. The quality of the EBV-specific DNA probe was verified by FISH on EBV+ BL-CL Namalwa, which is known to bear only one EBV-IS at the chromosomal region 1p35 [20]. Negative controls were performed on EBV-negative BL-CLs CA-46 and CW698 (Figure 1).

The analysis was performed using fluorescence microscopy. Fifteen well-conserved and complete metaphases counterstained with DAPI were analyzed in all BL-CLs except Naliaka. In this latter, eight metaphases were analyzed. Inverted DAPI counterstaining to obtain a banding pattern similar to G-banding was used for assignment of the EBV-IS to a chromosomal band or cytoband (resolution at 150–300 band level). The metaphases of each BL-CL were analyzed concerning the viral integration site, the number and recurrence of EBV-IS, and episomal form. Integration of EBV into the host genome was defined by the presence of symmetrical doublet hybridization signals (DS) at the same chromosomal loci on both sister chromatids and the corresponding cytoband was assigned as integration loci [12,17,20]; single hybridization signals with weak intensity were characterized as episomal viral location [12,20].

### 2.4. Statistical Analyses

The preferential regions for EBV integration were analyzed considering the Poisson distribution based on the number of EBV-IS by metaphase and by cell line, respectively. The statistical analyses for the correlation of EBV-IS into common and rare fragile sites (CFS and RFS, respectively) as well as into the breakpoints of the BL-CL structural variants were performed using a Binomial Exact test. The expected EBV integration was computed assuming a random uniform distribution of the BL-CL breakpoints, CFS, and RFS respectively in the 368 cytobands defined [33]. The cytogenetic and molecular cytogenetic data of the same BL-CL previously reported by us were used to determine the breakpoints due to structural variants present, i.e., translocations, duplications, deletions, inversions, etc [32]. For calculation, in the regions of conflict where the integration site was spanning several cytobands, the integration site was assigned to the most probable band, based on the integration in other cell lines.

## 3. Results

In this study, we performed a molecular cytogenetic mapping of EBV integration on 20 EBV+ BL-CLs by FISH with a home-brewed EBV-probe [23]. The presence of the EBV genome in BL-CLs was confirmed in all EBV+ BL-CLs analyzed by PCR. For FISH experiments, EBV+ BL-CLs Namalwa and BL60 were used as quality control, as cytogenetic integration data of these BL-CL are available (Table 1). Namalwa presented bright DS of the EBV probe at the expected chromosomal localization on 1p35, showing an extremely robust integration pattern of EBV after years of cultivation in different in vitro conditions. BL60 showed a singular EBV-IS on a derivative chromosome 19 der (19) near the breakpoint of a translocation, as previously described [13]. However, the translocation partner was found to be chromosome 17 in der (19)t(17;19), which is not in line with the previously published chromosome 11 as an affected chromosome in der (19)t(11;19) [13]. This discrepancy most likely relies more on the specificity of multicolor-FISH that we applied to identify structural aberrations, comparing to the classical cytogenetics; it is also conceivable that in the frame of clonal evolution, der (19)t(11;19) was the target of further structural change within chromosome 17. In both scenarios, the EBV-IS remained on the der (19) near the breakpoint.

Hybridization signals emitted by episomal viral DNA forms were detected in all EBV+ BL-CL but not in Namalwa. Using FISH, episomal and integrated forms of the viral genome can reliably be distinguished from each other and from interfering noise, as it has been endorsed by numerous previous studies [12,14,15,16,17,21,23,24,25,26,27]. EBV-negative BL-CLs CA-46 and CW698 were used as negative controls and showed no hybridization signals for the used EBV-probe (Figure 1).

We observed considerable differences in the number and localization of DS between the BL-CLs. According to the EBV-IS pattern, we distinguished three different behaviors that we used to group the BL-CLs (Table 2). Group 1 was defined by one recurrent EBV-IS in 100% of the metaphases analyzed, without the presence of concomitant non-recurrent EBV-IS, and comprised of four BL-CLs (BL-60, Naliaka, PA-682, and Seraphine), in addition to the well-known BL-CL Namalwa.

Group 2 was the largest group in this study, comprising of 13 BL-CLs, which exhibit at least one recurrent EBV-IS accompanied by multiple non-recurrent EBV-IS. The number of recurrent EBV-IS per BL-CL ranged from one to eleven, showing frequencies between 13% and 100%. Highly recurrent EBV-IS (frequency 67~100% of the metaphases) were seen in AG876, LY-67, Maku, and Switzer (Figure 1). The remaining nine BL-CLs of this group did not display any high recurrent EBV-IS (frequency < 40% of the metaphases).

Group 3 includes three BL-CLs (EB-1, LY-47, and Salim Mwalim), which showed exclusively non-recurrent EBV-IS. In about 50% of metaphases, no symmetrical doublet hybridization signals for EBV were detected in this group of BL-CLs.

### 3.1. Distribution of EBV-IS in the Whole Collective of BL-CL

**Figure 2:**
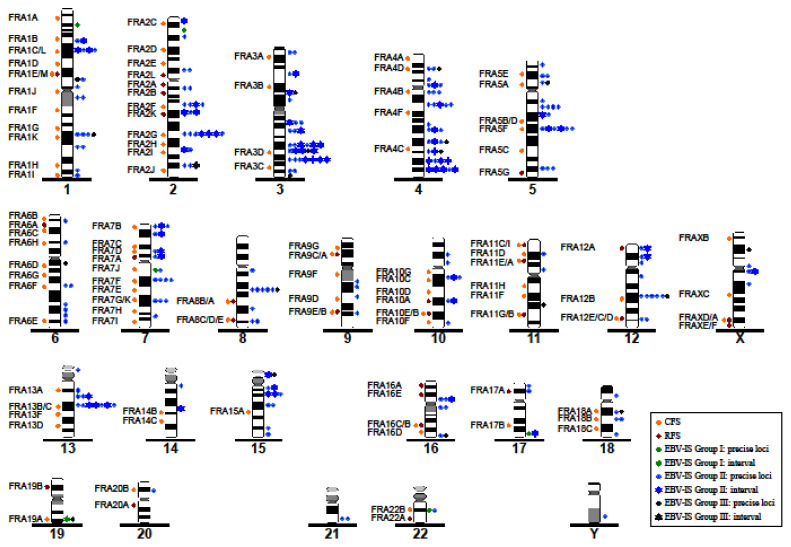
The distribution of EBV-IS and CFS/RFS at the chromosomal level. Common and rare fragile sites (CFS and RFS, respectively) are depicted at chromosomal cytobands on the left side of the chromosome ideograms, and on the right side, EBV-IS observed in our study are illustrated. The green dots represent EBV-IS of group 1 BL cell lines, blue of group 2, and black of group 3. In case of conflict, when the integration site was spanning several cytobands the integration site was assigned to the most probable band based on the integration observed in other BL cell lines. Conflict cases are indicated as a star and their colour reflects the equivalent BL-CL group as indicated above.

A total of 459 DS were detected in 293 metaphases from 21 BL-CL, including Namalwa, analyzed in this study. Four hundred and fifty-three DS could be precisely assigned to a cytoband and six DS only to a chromosomal arm (Table 2). EBV-IS were distributed on all chromosomes (Figure 2). The most commonly affected chromosomes by EBV-IS (>30 DS) were chromosome 2 (59 DS), chromosome 4 (55 DS), chromosome 13 (51 DS), chromosome 7 (46 DS), chromosome 1 (41 DS), and chromosome 3 (36 DS), which make a total of 288 (63%) of the identified DS. Rarely affected were chromosomes 9, 11, 14, 20, 21, and the sex chromosomes with five or less DS per chromosome. An accumulation of EBV-IS (>30 DS) was detected on the chromosomal arms 4q (44 DS), 2q, and 13q (both 36 DS). No EBV-IS were visualized on the chromosomal arms Yp, 8p, 9p, 14p, 20q, 21p, and 22p. In the remaining chromosomal arms, less than 30 DSs were observed.

Next, we investigated the distribution of EBV-IS at the chromosomal band level. A total of 119 out of 368 cytobands (32%) were the target of integration for the EBV genome. A total of 293 metaphases were studied across 21 BL-CL. By metaphase, statistically highly significant EBV-IS (*p* < 0.0001) were located on 13q21 (33 metaphases); 19q13 and 22q12 (16 metaphases); 1p35, 2p23, 2q31, 7p22, and 13p11 (15 metaphases); 3q26 and 7q21 (13 metaphases); 2q21, 4q21, and 4q34 (12 metaphases); 15q14, and 17q25 (11 metaphases); as well as 1q31 and 12q21 (eight metaphases). Further statistically significant EBV-IS (*p* < 0.001) were located on 4p12, 5q21, 7q11, and 8q21 (seven metaphases); as well as 4q28 (six metaphases); followed by (*p* < 0.05) 4q32 and 10q21 (five metaphases); and 1p31, 2p12, 3q28, 4q12, 5q13, and 7q31 (four metaphases).

Since the distribution of EBV-IS in the BL-CL by metaphase was unequal, ranging from only one recurrent EBV-IS in all metaphases in group 1 to up to 11 non-recurrent EBV-IS in a different number of metaphases in groups 2 and 3, we calculated the distribution of the EBV-IS by BL-CL by counting each EBV-IS only once per BL-CL. The number of statistically highly significant EBV-IS reduced to 13 (*p* < 0.0001) at the following cytobands: 2q31, 3q26, and 13q21 (7 BL-CL); 5q21, 8q21, and 12q21 (6 BL-CL); 4q21 and 4q34 (5 BL-CL, *p* < 0.001); as well as 1p31, 1q31, 4q28, 5q13, and 7q21 (4 BL-CL, *p* < 0.01).

Taken together, the distribution by metaphase and by BL-CL, we observed 13 statistically significant EBV-IS at the chromosomal bands 1p31, 1q31, 2q31, 3q26, 4q21, 4q28, 4q34, 5q13, 5q21, 7q21, 8q21, 12q21, and 13q21 in BL-CLs. The remaining 17 statistically significant EBV-IS by metaphase did not overlap in the analyses by BL-CL. Interestingly, none of the 13 recurrent EBV-IS overlapped with the highly recurrent EBV-IS (67~100% of the metaphases) present in the BL-CL of group 1 or group 2, with the exception of 7q21 and 13q21, both in LY-67 of group 2.

### 3.2. Comparison between EBV-IS in BL-CLs and LCLs

Further, we investigated whether the EBV integration patterns detected in our study are BL specific by comparing the identified EBV-IS with the published data of 24 well-documented EBV-transformed LCLs (Table 1) [14,20,21,22,24,25,26]. In LCLs, 73 cytobands displayed an EBV-IS, of which 38 (52%) coincide exactly with the EBV-IS visualized in the analyzed BL-CL. If only the 13 statistically significant overlapping regions between EBV-IS by metaphase and by BL-CL are taken into account, six of them are localized exactly at the same cytobands (1p31, 1q31, 3q26, 4q34, 5q21, and 13q21) and two (2q31 and 4q21) at the immediately proximal cytoband. Taken together, these data strongly indicate that EBV integration follows a pattern of integration and occurred preferentially at the common cytobands 1p31, 1q31, 2q31, 3q26, 4q21, 4q34, 5q21, and 13q21 in both BL-CL and LCL, and at 4q28, 5q13, 7q21, 8q21, and 12q21 in only BL-CL.

### 3.3. Correlation between EBV-IS and Genetically Unstable Regions

**Figure 3:**
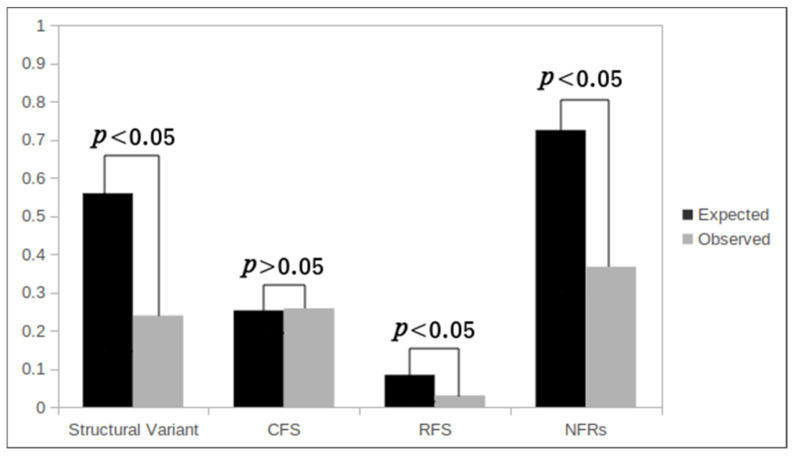
Summary of the EBV-IS distribution. Distribution of 453 out of 459 EBV-IS, that could be precisely assigned to a cytoband, in the chromosomal breakpoints (structural variants), in the common fragile sites (CFS), in the rare fragile sites (RFS), and in non-fragile sites (NFR). The expected EBV-IS (grey bars) and the observed EBV-IS (black bars) ratios of integration of EBV were calculated assuming a uniform, random distribution. *p* values were calculated by a binomial exact test.

CFS are chromosomal regions prone to break during DNA replication inhibition, under specific cell culture conditions. Up to now, 88 official CFS have been described in the human genome together with 31 rare fragile sites (RFS) [34], a list that has been increased with new CFS (Figure 2). To determine whether our identified EBV-IS coincide with CFS and/or RFS, we analyzed the colocalization of DS with known CFS and RFS separately. A colocalization of an EBV-IS on a CFS or RFS was seen in 42 specific cytobands (33.87%). No significant integration of EBV at the CFS was observed (*p* > 0.05). For the RFS, the expected EBV integration was higher than the observed integration with a statistical significance (*p* < 0.05) (Figure 3).

A further consequence attributed to viral integration into host genomes is related to the induction of chromosomal instability and chromosomal rearrangements. Although viral DNA integration was detected at chromosomes involved in structural variants in five BL-CLs (BL60, Maku, JBL-2, JI, and Salim Mwalin), only two EBV-IS (13p11 in Maku and 8q24 in JBL-2) were located at a breakpoint region (Table 2 and Table S1). To further investigate the association between the integration of EBV and chromosomal rearrangements, we obtained the breakpoints from the karyotypes of all 20 BL-CLs and Namalwa from our previous study [32], in which we exhaustively characterized them by G-banding/M-FISH and FISH, and performed a statistical analysis of the EBV-IS and breakpoint distribution. The expected integration at the chromosomal breakpoints was higher compared to the observed integration with a statistical significance (*p* < 0.05) (Figure 3).

### 3.4. Pattern of Integration of Statistically Significant EBV in the Chromosomal DNA Sequences

Preference of EBV for integration into G-bands was known for LCLs [11,12,13]. G-bands correspond to late replicating bands during the S-phase and are gene-poor regions [34]. G-bands contain AT-rich DNA sequences and a concentration of the long interspersed nuclear element L1 (LINE1). In contrast, the early replicating T-/R-bands concentrate a high density of Alu sequences, a clustering of CpG islands, and, accordingly a gene-richness [35]. In line with those previous observations in LCLs, all but three (2q31, 4q21, and 5q13) 13 preferential EBV-IS were located at G-positive cytobands, of which eight were visualized on the gene poorest CpG regions of the human genome (1p31, 1q31, 3q26, 5q21, 7q21, 8q21, 12q21, and 13q21) [36]. Therefore, EBV shows a non-random preference to integrate precisely into cytobands poor in genes and CpGs and are enriched in LINE1 repeat regions.

Interestingly, we observed another pattern of EBV integration in all BL-CLs of group 1. None of their EBV-IS was visualized at any of the 13 aforesaid recurrent G-bands. Moreover, all EBV-IS were located on the CpG-richest R-positive cytobands [36]. This was also true for the highly recurrent EBV-IS of group 2 cell lines Switzer (2q21) and AG876 (7p22). All eight of these EBV-IS, located at the cytobands 1p35, 2p23, 2q21, 7p22, 7q11, 17q25, 19q13, and 22q12, were statistically significant by metaphase but not by BL-CLs, and accounted for 27% of statistically highly significant EBV-IS by metaphase.

## 4. Discussion

EBV infection plays an important role in the pathogenesis of BL and other human neoplasias, however, the precise mechanisms of the viral infection in the pathogenesis of cancer are still unclear [8]. Numerous studies demonstrate the coexistence of episomal forms and the integration of the EBV-DNA in the human genome of carcinomas, lymphomas, and LCL [11,12,13,14,15,16,17,18,19,20,21,22,23,24,25,26,27,37]. Whether the integration of EBV-DNA into the human genome follows a specific integrational pattern has been the subject of studies since the 1980s. Several cytogenetic studies, mainly in LCL, have provided enough evidence of a non-random pattern for the integration of EBV, contradicting how it was once thought to be random [12,14,15,16,17,18,19,20,21,22,23,24,25,26,38]. In this study, we used FISH to map the EBV-IS in 20 previously cytogenetically characterized EBV+ BL-CLs [32], in addition to the reported BL-CL Namalwa [20]. This study represents the largest compendium of EBV-IS analyzed in BL-CL.

We found that all EBV+ BL-CLs fulfilled the criteria for the EBV integration. An integration of the EBV-DNA, defined as symmetrical doublet hybridization signals, was seen in all chromosomes but the distribution pattern and frequency notably differed among the BL-CLs. Based on the frequency of the EBV-IS, we clustered the BL-CLs in three groups; group 1, which exhibited unique recurrent EBV-IS in all metaphases, group 2 with at least one recurrent EBV-IS accompanied with multiple non-recurrent EBV-IS, and group 3 with exclusively multiple non-recurrent EBV-IS. An accumulation of EBV-IS was seen over chromosomes 1–4, and chromosomes 7 and 13, with 63% of integration loci found at those chromosomes. Similar observations regarding the number and chromosome localization of EBV-IS were reported in LCLs [14,20,21,22,24,25,26]. Episomes co-existed with the integrated form in all BL-CLs with the exception of Namalwa, which is keeping with the previous reports summarized in Table 1.

We further investigated the distribution of the EBV-IS on chromosomes of BL-CLs and compared it with published data on LCL. This comparison found that 52% of the viral integrations in BL-CLs and LCLs occurred on the same cytobands. The distribution of EBV-IS in BL-CL revealed a non-random integration pattern into 13 statistically significant cytobands, of which eight bands coincide with sites of viral integration in LCLs [14,20,21,22,24,25,26]. The direct comparison of our data to published data on BL or BL-CL was not possible because the majority of the cytogenetic data of EBV integration in BL reported only localization at chromosomal arms and not at cytobands. Nevertheless, the cytoband 13q2 and the chromosomal arms 3q, 4q, and 7q were also reported as preferential targets in the Jijoye BL-CL and 1q31, 5q2, 13q2 in the LCL-derived cell line P3HR-1 [21]. Similarly, in the BL-CL Raji, 64% of all EBV-IS were visualized at chromosomal regions 1q, 2q, 4q, and 7q [23] and among others at the cytobands 1q31 and 7q21 [21]. These results evidence an integration of the EBV-DNA in a non-random fashion in both BL-CL and LCL into the cytobands: 1p31, 1q31, 2q31, 3q26, 4q21, 4q34, 5q21, and 13q21. In addition, the cytobands 4q28, 5q13, 7q21, 8q21, and 12q21 are also statistically significant integration sites of EBV in BL-CL.

The consequences attributed to the viral integration of EBV and other viruses into the human genome are still not deciphered. The ability of some viruses to integrate into the human genome could be aided by the inherent chromosomal instability of the tumor cells, which in turn could exacerbate the genomic instability in the host cells [11,39]. It is therefore not surprising that fragile sites (CFS and RFS), known sites of increased chromosomal instability, have been identified as preferential regions for viral integration [40]. Despite the intrinsic genomic instability, CFS/RFS are evolutionarily conserved in all individuals and CFS frequently correlate with sites of genomic rearrangements in cancer [40]. A recent systematic study of viral integration sites in the human genome of five DNA oncoviruses, including EBV, reported that 33.9% of hepatitis B virus (HBV)-IS, 37.5% of human papillomavirus (HPV)-IS, 42.2% of HIV-IS, 34.6% of human T lymphotropic virus type 1 (HTLV-1)-IS, and 35.2% of EBV-IS colocalized at CFS/RFS [41]. This is in line with our findings that show an EBV integration in a CFS/RFS in 33.87% of the EBV-IS. A statistical correlation between viral integration sites and CFS/RFS has been reported for HBV and HPV [14,42], and for EBV in NPC and gastric carcinoma [29], however, we did not observe this in our collective of BL-CLs. Likewise, EBV-IS were not significantly enriched at the breakpoints of the chromosomal rearrangements in the BL-CL analyzed. Using WGS, numerous IS were found to be positively correlated with chromosome structural variations and copy number structural variations in BL-CL Raji indicating a non-random integration pattern [12]. These contradictory results probably rely on different sensitivity of cytogenetic and molecular cytogenetics compared to the WGS and the lack of more WGS data in BL. These data suggest that the integration of EBV in BL might be specific and differs from that observed in other EBV-associated cancers. Consistent with this observation, a phylogenetic analysis of EBV isolates derived from NKTCL samples has revealed a convergence respecting the geographic origin [28]. Moreover, EBV-genomes and -transcriptomes were closely clustered in a disease-specific manner that deviated apart from clusters of other EBV-associated cancers [28].

The human genome organization is non-arbitrary, interphase chromosomes are highly organized in chromosome territories with specific cytobands occupying their own compartments in the cell nucleus. As the gene density varies interchromosomally, the gene-dense and less-gene-dense regions vary intrachromosomally as well [35]. Up to 63% of the EBV-IS observed in our study were located on gene-poor chromosomes, including chromosomes 4 and 13, which have the lowest gene density in the human genome. Accordingly, we also found a preferential integration of the statistically significant EBV-IS into G-positive cytobands, which are gene- and CpGs-poor but rich in LINE1 repeat- and AT-regions. A similar distribution was observed for LCLs [11,12,13] and, more recently, has been confirmed by high-throughput sequencing in NPC, gastric carcinoma, and NKTCL [28,29] and other DNA oncoviruses such as HBV [31]. Interestingly, this affinity of EBV to gene-poor chromosomes has been also observed in the latent episome form of EBV in BL-CLs and LCLs [43]. Using Hi-C methods, Moquin and collaborators provided evidence that EBV latent episomes preferentially associate with AT-rich regions of chromatin distant from the transcription start sites of the large gene-poor chromosomes 2 to 5, this interaction being especially strong for chromosomes 4 and 13 [43]. The precise mechanisms by which the EBV genome integrates into the human chromosomes are unknown. Our data together with published data reveal a clear preference of EBV-DNA to be associated with gene-poor chromosomes and regions, especially with chromosomes 4 and 13, in the episomal and integrated form [14,21,22,23,24,25,26,43]. Moreover, one could speculate that the localization of the EBV episomes at the human chromosomes predetermine the sites of posterior chromosome integration of the viral DNA at precise cytobands [43] through mechanisms that have to be further investigated in future experiments.

However, we observed a different pattern of integration for the EBV-IS of the BL-CLs of group 1. These EBV-IS were unique of each BL-CL, not accompanied by other EBV-IS, and did not overlap in localization with the statistically significant 13 cytobands of integration of EBV or episomal association. Moreover, these EBV-IS were located on the CpG-richest R-positive cytobands 1p35, 2p23, 7q11, 17q25, 19q13, and 22q12 [35]. This preferential integration of EBV into a few functional genomic regions including CpGs could be associated with the disruption of genes, as it has been described for other DNA viruses such as HBV and HPV [31,42], and might represent a different tumorigenic mechanism of EBV integration. For instance, the BL-CL NAB-2 would perfectly fit in this model, since it shows a unique EBV-IS on the CpG-rich cytoband 2p13 between *REL* and *BCL11A* that lead to an increased expression level of the *REL* oncogene [19]. Whether the pattern of EBV integration in BL-CL is comparable with that of primary BL samples or the acquisition of EBV integration results during the long-term culturing of the BL-CL will be aimed in future investigations.

## 5. Conclusions

Collectively, our data provide evidence for a dichotomous pattern of EBV integration in the BL-CL analyzed in our study. Mostly EBV-DNA integrates into multiple genome localizations in BL-CLs following a non-random integration pattern into specific gene-poor G-positive cytobands, which overlap with the pattern reported in LCLs. Moreover, these G-positive cytobands are closely connected with the preferred chromosomal bands of the EBV episomes. However, in a small subset of BL-CL, EBV integrates into a singular cytoband, located in a gene-rich R-positive genomic region, and most likely linked to a deregulation of the genes in the vicinity of the EBV-IS. Our results provide new insights into the mechanisms of EBV integration in BL-CLs at the chromosomal level. Future studies using WGS will deepen this data at the molecular level and will help to understand the pathogenic role of EBV integration and the development of BL.

## Figures and Tables

**Table 1:** EBV integration sites reported in 8 BL-CLs, 24 EBV-transformed lymphoblastoid cell lines (LCLs), and 1 Hodgkin lymphoma cell line at chromosomal band resolution level.

Cell Line	Origin	Chromosomal Location	Gene Involved	Reference
BL36	BL *	11p15 + Episomal form	Unknown	[12]
BL60	BL	der (19)t(11;19) + 1 Episomal form	Near the breakpoint	[13]
BL137	BL *	1p34 + Episomal form	Unknown	[12]
EB-2	BL	1p, 1q, 3p, 3q, 4p, 4q, 5p, 5q, 6p, 6q, 8p, 8q, 11q, 12q, 13q, 14q, 19p, 10q, 21p + Episomal form	None	[14]
				[15]
				[12]
				[16]
				[17]
NAB-2	BL	2p13	Between *REL* and *BCL11A*	[18]
				[19]
Namalwa	BL	1p35	*MACF-1*	[20]
Raji	BL **	Preferential: 1q, 2q, 4q, and 7q Further: 1p, 3p, 3q, 5q, 6q, 7p, 9q, 11p, 14q, 15q + Episomal form 1q31, 3q13, 7q21, 7q3, 8q23, 21q	*BACH2*/6q15	[21]
				[22]
				[23]
Jijoye	BL **	Preferential: 13q2 Further: 3p, 3q, 4p, 4q, 5p, 6p, 6q, 7q, 8q, 11p, 18q, 21q + Episomal form	Unknown	[21]
P3HR-1	LCL (derived from Jijoye)	1q31, 5q2, 13q2, 21q1 + Episomal form	Unknown	[21]
IB-4	LCL	4q25 + Episomal form	RP11-119H12	[20]
				[22]
n = 3	LCL **	1p31, 1q31, 2q32, 3q13, 6q24, 7q31 (in all 3 LCL) + Episomal form	Unknown	[24]
n = 14	LCL **	Preferential: 5p14 (in all 14 LCLs); 2p22 (in 13); 1p31, 1q43, 3q28, 4q13, 5q12, 11p15 (in 12); 1q31, 2q32, 4p15, 5q34, 10q26, 12p12 (in 11); 1p36, 3p24, 4q26, 6q24, 10q21, 13q13 (in 10) Further: up to 60 integration sites in total + Episomal form	Unknown	[14]
n = 4	LCLs ***	1p31, 1q31, 4q22~25, 5q21, 13q21 and 14q21 (in all); 2q22~24, 3q11, 3q22~24, 3q26, 5q32~34 and 14q31 (presumably in all) + Episomal form	Unknown	[25]
ATL9/g	LCL	1p31~32, 1q31, 2p12, 4p11, 5q21, 11p13, 11q22, 13q2, 16p13, 18q12 + Episomal form	Unknown	[26]
AM-HLH	HL	Multiple copies on chromosome 20 of a der(21)t(19;20;21)	Unknown	[27]

LCL, in vitro immortalized human lymphoblastoid cell lines; HL, Hodgkin lymphoma; * Further double signal/integration sites reported but localization not given; ** Preferential integration sites; *** Not distinguished between EBV doublet or single signals.

**Table 2:** Chromosomal distribution of the recurrent and non-recurrent EBV-IL in analyzed BL cell lines.

	BL-CL	Frequency: Recurrent IS	Frequency: Non-Recurrent IS
Group 1	BL60	100%: 19 on der (19)t(17,19) (near the breakpoint)	None. Episomal.
	Naliaka *	100%: 17q25 75%: 7q11	None. Episomal.
	Namalwa	100%: 1p35	None.
	PA-682	100%: 22q12	None. Episomal.
	Seraphine	100%: 2p23	None. Episomal.
Group 2	Maku	100%: 13p11 on der (13)t(3;13) ins (3;13)	2q31, 3p25, 3q24, 4p12, 4q31, 5p14, 8q21. Episomal.
	AG876	80%: 7p22 53%: 13q21	1q12, 3q12~13, 3q24, 3q28, 4q33~35, 5q13, 7q31, 8q11, 10p11, 10q23, 10q26, 13q14, 16p11. Episomal.
	LY-67	80%: 13q21 67%: 7q21 20%: 12q21 13%: 3q26	1q43, 3p, 3q13, 4p13, 4q21, 5q13, 5q21, 6q26, 10q21, 15p. Episomal.
	Switzer	67%: 2q21 33%: 2q31 27%: 1q31, 3q26 20%: 4q31~34 13%: 1p32, 13q21~31	2p12, 2q36, 4q12, 5q14~23, 5q34, 7p13, 7q11, 7q34, 8q21, 15q21, 18p11.2. Episomal.
	BL16	40%: 4q21 33%: 4q32 20%: 3q28, 10q11~21 13%: 1p11, 2p22, 2q12, 3q24~26, 7q31, 15q21, 16q11	2q21, 2q31, 3p25, 3q21, 4p15, 4q12, 4q26, 5q21, 5q34, 9q13, 9q22, 9q33, 10p12, 12q21, 14q11, 15q11, 16p11, 18q23. Episomal.
	JBL-2	33%: 15q13~15 20%: 13q21~31 13%: 1q31, 8q24 on der (8)t(2;8), 11p14, 17p12	Yq12, 1q44, 4p15, 4q26, 5q12, 5q13, 6q23, 7p12, 7q21, 8q21 on t(2;8), 8q23, 11p11, 12p12, 12q21, 13q13, 15q25. Episomal.
	Rael	33%: 4q32~34 20%: 2p11~12, 4q21~24, 4q26~28 13%: 4p12	Xq10~11, 1p32~34, 2q22~24, 2q31, 5q13, 6q21, 6q24, 7p22, 12q13, 13q14, 14q22~23, 16q10~11, 18q21. Episomal.
	Akuba	27%: 4p11~12 20%: 13q21 13%: 1q32, 4q12, 8q21, 15q13~14	Xq21, 1p31, 2q22, 3q21, 3q24, 3q26, 4q21, 5p13, 6p21, 6q12, 6q21, 7q21, 12p13, 12q24, 16p11~12. Episomal.
	JI	27%: 15q11~14 13%: 2q21~31, 12p12~13, 12q24	1q13 on der(6)t(1;6), 3q25, 4q25, 5q21~23, 7p21, 6p24 on der(6)t(1;6), 8q24, 12q21, 15q26, 17p13, 21q22. Episomal.
	Silfere	20%: 2q31~33, 17q22~25 13%: 3q21~26	1p13, 1p21~31, 3p21, 4q28, 5q14, 5q21, 5q34, 7q31, 10q21, 12p11, 12q21, 16q24, 18q21, 20p12, 21q22. Episomal.
	Solubo	20%: 13q14~22 13%: 4q34, 7p11~13	1p21~31, 2pter, 2q21, 2q33, 5p15, 9q21. Episomal.
	BL18	13%: 2q24~32, 3p13~21	Xp10, Xq10, 1p31, 1q31, 2q36, 3q24~26, 4q21, 6q25, 7q21, 8q21, 10q23~25, 22q12. Episomal.
	LY-91	13%: 5q21, 7p21~22, 13q21~31, 15p11~13	1q32, 2p24~25, 2q12, 3p12, 3q13, 3q26~27, 4q28, 4q32~35, 5p14, 9p, 12p, 18q12. Episomal.
Group 3	EB-1	none	2q34~36, 3q29, 4q28, 5p13, 12q21, 16q24, 19q13. Episomal.
	LY-47	none	1p13, 3p13, 3q25, 4p15, 11q22, 15p11, 18q12. Episomal.
	Salim Mwalim	none	Xp21, 1q31, 4q31, 7p on dup(7)(q11q22.3), 8q21. Episomal.

* 15 metaphases were analyzed in all cell lines but Naliaka. In the latter, eight BL-CL metaphases were analyzed.

## Data Availability

Not applicable.

## Supplementary Materials

The following supporting information can be downloaded at: https://www.mdpi.com/article/10.3390/v14010086/s1, Table S1: EBV + Burkitt-Lymphoma cell lines (BL-CLs) used in this study including their published karyotypes.
