# Supplementary material for: Non-Random Pattern of Integration for Epstein-Barr Virus with Preference for Gene-Poor Genomic Chromosomal Regions into the Genome of Burkitt Lymphoma Cell Lines
Source: Viruses. 2022 Jan 4;14(1):86. doi: 10.3390/v14010086 (PMC8781420; doi:10.3390/v14010086)
Supplement: Supplementary file 1 [file viruses-14-00086-s001.zip › viruses-1485220-supplementary.pdf]

viruses

MDPI

Supplemental Table S1. EBV + Burkitt-Lymphoma cell lines (BL-CLs) used in this study including their published karyotypes [32].

BL-CL	Type/ethnic GrouEp	Sex/Age	Consensus Karyotype Based on the G-Banding, Multicolor-FISH and Metaphase FISH Results Previously Published
AG876	BL/ African	M/8	50,XY,t(8;14)(q24;q32),+9,+13,+15,dup(17)(?),+21
Akuba	BL/ African	n.a.	50,XX,t(8;22)(q24;q11),+12,+13,+19,del(19)(p11 or q11),+20/51,sl,+4/50,sl,der(X)t(X;13)(q24;q31.1),i(13)(q10)
BL16	BL/ African	F/5	46,XX,t(8;14)(q24;q32),der(13)dup(13)(q31.1q34)t(1;13)(q31.3;q34)
BL18	BL/north -African	M/3	46,XY,t(8;14)(q24;q32),der(11)t(3;11)(q2?5;q23)/49,XY,+3,+4,+7,t(8;14)(q24;q32)
BL60	BL/north -African	F/4	46,XX,der(3)dup(3)(q?13q?29)t(3;12)(q29;?),t(8;22)(q24;q11),del(17)(p11),der(19)t(13;19)(q31.1;p13 or q13),der(19)t(17;19)(?;p13 or q13)/46,sl,der(18)t(9;18)(?;q21)/48,sl,+7,+9/45,sl,−X,del(18)(q12)
CA-46	BL/American	n.a.	46,X,−Y,dup(1)(q31.3q11),dup(7)(q11q22.1),t(8;14)(q24;q32),der(13)dup(13)(q31.3q21)del(13)(q31.3q34),+16/46,X,sl,ider(13)(q10)dup(13)del(13)/47,sl,+der(7;13)(q10;q10),der(13)dup(13)del(13)×2
CW698	BL/ American	n.a.	46,XY,dup(1)(q31.1q12),der(2)t(2;13)(p2?5;q13),t(3;21)(p14;q22),der(8)t(8;14)(q24;q32),del(11)(q21),der(13)dup(13)(q33q22)t(5;13)(?q22;q33),der(14)t(7;14)(q11;p11),der(14)t(14;16)(p11;p11)t(8;14),del(17)(p11),der(18)t(18;21)(q23;q22)/46,idem,dup(1)(p31p34
EB-1	BL/African	F/9	47,XX,+X,t(8;14)(q24;q32),dup(11)(q?22q?23)/46,sl,−6/48~53,sl,+2,+7,+15/87~93,sl×2,der(X)t(X;1 or 7)(p22;?)×2,−6,−8,−11,der(11)t(7;11)(q11;q13),der(16)t(2;16)(?;p?),der(21)t(17;21)(?;?)
JBL2	BL/Japanese	M/29	47,XY,t(2;8)(p12;q24),+13
JI	BL/Caucasian	F/34	49,XX,t(2;8)(p12;q24),der(6)t(1;6)(q12;q13),+15,+16,i(16)(q10),+21/49,idem,−X,+13,der(16)?t(16;16)(p?;q?),−i(16)
LY-47	BL/African	M/n.a.	46,XY,+7,der(8)t(8;22)(q24;q11),der(10)t(10;16)(p15;?q11),del(11)(q21),der(16)t(1;16)(q12;q11),−22
LY-67	BL/African	M/8	47,X,der(Y)t(Y;20)(?;?),der(4)t(4;6)(q31;p21),der(6)t(4;6)t(6;15)(q21;q15),t(8;22)(q24;q11),del(11)(q23),+13,der(17)t(3;17)(q22;q23),der(18)t(7;18)(q34;q2?2)/47,X,der(Y)t(Y;20)(?;?),der(4)t(4;6)(q31;p21),der(6)t(4;6)t(6;15)(q21;q15),t(8;22)(q24;q11),del(11)(q23),der(11)t(3;11)(q22;q24),+13,der(18)t(7;18)(q34;q2?2)
LY-91	BL/African	F/7	46,XX,t(2;8)(p12;q24)
Maku	BL/African	M/n.a	46,X,der(Y)t(Y;16)(q12;q12),i(3)(q10),der(6)t(3;6)(p21;q23),t(8;22)(q24;q11),t(10;17)(p11;q23),der(13)t(3;13)(p12;p11)ins(3;13)(p21;q31.1q33)
Naliaka	BL/African	n.a.	46,X,?−X,t(8;14)(q24;q32),+12,der(12)t(12;13)(q15;q14),der(15)t(15;17)(q26;?)
Namalwa	BL/African	F/3	44~46,X,−X,dup(1)(q31.1q12),der(2)t(2;3)(q3?7;p21),del(3)(p11),der(3;21)(q10;q10),der(3)t(3;5)(q2?5;q1?5),der(3)(3pter→3q2?5::5q1?5→5?q35::3p21→3pter),der(3)(3pter→3q?::5?::3q?→3q?25::5q1?5→5?qter),del(5)(q1?5),der(6)t(3;6)(p21;p23),+7,del(8)(p22),der(8)t(8;14)(q24;q32),der(13)t(13;13)(p11;q21.1)t(13;13)(q21.1q31.3;q31.3q34)dup(13)(q33.1q31.1),der(14)t(8;14)(p22;p11)del(14)(q11q13)t(8;14),der(15)t(3;15)(p21;p11),der(15;21)(q10;q10),+21[cp]
PA-682	BL/ Caucasian	M/30	46,XY,der(6)t(6;17)(q23;?q23),der(7)t(7;17)(p22;?),t(8;22)(q24;q11),der(13)t(13;13)(p11;q31.1)del(13)(q22q31.1),der(15)t(6;15)(?;p11),del(17)(q23),der(18)t(3;18)(q11;q21)/46,XY,idem,t(1;15)(p22;p11)

Rael	BL/African	n.a	44,X,?−Y,dup(8)(q?),t(8;14)(q24;q32),del(9)(q22),der(9)del(9)(p13)t(9;17)(q13;q21),der(15)t(9;15)(q22;p11),−17,i(17)(q10),der(18)t(18;20)(q23;q11)
Raji	BL/African	M/12	46,XY,der(4)t(1;4)(p?34;q35),+8,der(8;8)(q10;q10)t(8;12)(q22;?)t(8;14)(q24;q32),del(9)(p23),der(14)t(8;14)
Salim Mwalim	BL/African	n.a.	46,XY,der(6)t(3;6)(?q27;q27),dup(7)(q11q22.3),t(8;14)(q24;q32)
Seraphine	BL/African	F/7	46,XX,t(8;14)(q24;q32),der(16)t(1;16)(q12;q11)/46,idem,der(9)t(6;9)(q?;q34)
Silfere	BL/ African	F/6	46,XX,der(6)t(1;6)(q32;q23),t(8;14)(q24;q32)
Solubo	BL/ African	n.a.	48,XY,+7,t(8;22)(q24;q11),del(11)(q23),der(18)t(7;18)(q34;q21),+20/47,idem,−Y/45,X,−Y,del(7)(q34),t(8;22)(q24;q11),del(11)(q23),der(18)t(7;18)(q34;q21)
Switzer	BL/American	M/16	46,XY,t(8;14)(q24;q32)

n.a. not available; M: male; F: female.
